# Efficacy of Liposomal Nystatin in a Rabbit Model of Cryptococcal Meningitis

**DOI:** 10.3390/jof10080520

**Published:** 2024-07-26

**Authors:** Charles D. Giamberardino, Wiley A. Schell, Jennifer L. Tenor, Dena L. Toffaletti, John R. Perfect

**Affiliations:** Department of Medicine, Division of Infectious Diseases, School of Medicine, Duke University, Durham, NC 27710, USA; charles.giamberardino@duke.edu (C.D.G.);

**Keywords:** Cryptococcus, cryptococcal meningitis, fungal meningitis, rabbit, polyene, nystatin

## Abstract

Cryptococcal meningitis (CM) causes significant global morbidity and mortality. Current therapeutic strategies rely on deoxycholated or liposomal forms of the polyene amphotericin B. Nystatin is also a polyene with broad-spectrum antimicrobial activity. Treatment with systemic nystatin has been limited by toxicity, which is a consistent challenge with polyene therapeutics. One mechanism to improve the toxicity is usage of a liposomal form of the active agent. Previous data from a murine candidemia model indicated that liposomal nystatin may be an effective antifungal drug formulation. Since the rabbit model of CM is a highly predictive preclinical system for evaluating antifungal therapeutics, we tested the effectiveness of two doses of daily liposomal nystatin, 3 and 8 mg/kg in the rabbit model of CM. Treatment with liposomal nystatin in this model did not reduce the fungal burden in the cerebrospinal fluid. A subsequent clinical trial also did not find activity in a human population. These data indicate that liposomal nystatin in the current form and at the tested dosages is not an effective therapy for CM. The data provide further evidence for the predictive power of the rabbit model of CM as a vital preclinical system for testing novel antifungal therapeutics for CM.

## 1. Introduction

Cryptococcal meningitis is a disease which occurs when the yeast cells of several *Cryptococcus species* invade the central nervous system. This entry results in meningoencephalitis, as the yeasts localize in both the subarachnoid space and brain tissue, along with its ability to increase intracranial pressure, which in turn causes severe headaches and neurological symptoms. If untreated, the infection is routinely fatal. Currently, the highest disease burden is in immunosuppressed populations, such as AIDS patients, particularly in resource-limited areas of southern Africa. However, *Cryptococcus species* can also infect immune replete populations and cause disease in otherwise healthy individuals. Current therapies are effective, but mortality still hovers around 20% even in the most ideal treatment scenarios [1] and approximately 40% with only access to fluconazole [2,3].

The current antifungal regimen for CM consists of three phases: induction with a combination of amphotericin B and flucytosine for two weeks, then high-dose fluconazole for two months, and then a lower dose of fluconazole for six months to one year. Liposomal versions of amphotericin B are the preferred polyene as they have lower toxicities and can even be given as a single dose during the induction phase, as opposed to the daily intravenous dosing with the deoxycholated form [4]. The deoxycholated form of amphotericin B causes significant nephrotoxicity; thus, clinical chemistries must be closely monitored during dosing, which can be a challenge in some healthcare settings. Unfortunately, the liposomal form, which is less toxic, can be more difficult to acquire and more expensive.

Nystatin is a polyene with good, consistent in vitro activity against *Cryptococcus* with an MIC_90_ = 8 µg/mL when tested against 20 strains of *C. neoformans* [5]. However, intravenous nystatin can be acutely toxic, which limits its use in a clinical setting [6]. The liposomal form was developed to improve the distribution and reduce its toxicity [7]. In a murine model of candidemia, five doses of liposomal nystatin resulted in the survival of all infected mice up to 60 days post infection [8]. In a neutropenic mouse model of invasive aspergillosis, liposomal nystatin given intravenously daily for five days resulted in clearance of the mold from the liver and kidney [9]. Furthermore, in a neutropenic rabbit model of pulmonary aspergillosis, liposomal nystatin given either at 2 mg/kg/day or 4 mg/kg/day reduced the fungal burden and prolonged host survival [10]. Finally, in a study of disseminated candidemia in neutropenic rabbits that were treated with 4 mg/kg/day of liposomal nystatin, the fungal burdens were below the limit of detection in all organs examined. These results were similar to the positive control animals in the experiment, which were treated with amphotericin B deoxycholate at 1 mg/kg/day [11]. Given its efficacy against *Candida* and *Aspergillus* in murine and lagomorph models, its low toxicity, and its consistent in vitro activity against *C. neoformans*, we hypothesized that daily intravenous liposomal nystatin would be effective in a rabbit model of CM. The rabbit model allows for repeated measurements of fungal burden within the central nervous system compartment in individual immune suppressed animals and thus enables the assessment of the efficacy and clearance rate of a candidate antifungal for CM. Here, we present data showing the poor efficacy of both 3 mg/kg and 8 mg/kg of liposomal nystatin when compared to daily amphotericin B in this model. This negative result matches the preliminary negative results from a human clinical trial of liposomal nystatin for CM and demonstrates the screening utility of the rabbit model for the initial evaluation of in vivo anticryptococcal efficacy of a drug for CM [12].

## 2. Materials and Methods

### 2.1. Animals

All experiments were conducted under a Duke University-approved Institutional Animal Care and Use Committee (IACUC) protocol. The studies used male New Zealand white rabbits weighing between 2 and 3 kg. Rabbits were immunosuppressed with daily injections of hydrocortisone acetate 5 mg/kg intramuscularly starting 1 day before the infection and continuing daily throughout the study period.

### 2.2. Organism

*Cryptococcus neoformans*, strain H99, was grown on Sabouraud dextrose agar. Individual colonies were selected and suspended in 0.9% saline. The suspension washed two times in PBS and then counted to ensure the inoculum was ~1 × 10^8^ cells of *C. neoformans* in 0.3 mL.

### 2.3. Inoculations and CSF Collections

Rabbits were sedated with ketamine (45–50 mg/kg) and xylazine (5–6 mg/kg) intramuscularly and then inoculated intracisternally with 0.3 mL of the inoculum. The rabbits were then recovered and the infection was allowed to progress for 48 h. Then, rabbits were sedated with ketamine and xylazine intramuscularly and ~0.5 mL of CSF was collected via aspiration from the cisterna magna on days 2, 5, 8, and 12 of infection. The CSF was serially diluted and cultured on Sabouraud agar plates with chloramphenicol for ~72 h at 30 °C, and the yeast colonies were counted to calculate the CFU/mL.

### 2.4. Measurement of Nystatin Levels

Blood was collected at 30 min or 4 h after the final dose or immediately after euthanasia, which was ~24 h after the final dose. The final CSF sample was collected ~24 h after the final dose, and then the rabbits were euthanized and brain tissue was collected. The concentrations of free nystatin in the blood, CSF, and brain tissue were quantified by the study sponsor. Any values below the limit of detection were recorded as 0 for analyses. The analytical methods were similar to previously published analyses [13].

### 2.5. Statistical Analysis

Data were analyzed in R (version 4.4). Plots were created using ggplot2 (v3.5.1) and emmeans (v1.10.2). The changes in total yeast burden through day 12 were analyzed using a Kruskal–Wallis or ANOVA followed by a post hoc T test or Wilcox signed-rank test with Bonferroni’s correction. Effective fungicidal activity (EFA) was calculated using the emmeans package and then the slopes of the models were extracted. The model had the parameters Log_10_ CFU/mL ~ Treatment*Day Post Infection + [0 + RabbitID|Day], where the fixed effects were the treatment and the day post infection, along with their interaction, and the random effects were the individual rabbit slopes, with a fixed intercept.

## 3. Results

**Figure 1:**
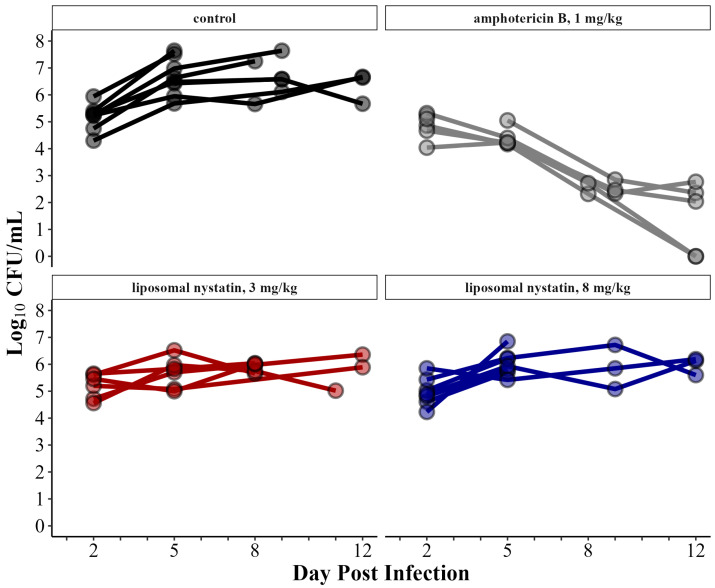
Data are from serial CSF collections from rabbits infected with *C. neoformans* and then either left untreated or treated with amphotericin B or liposomal nystatin. Each line represents data from a single rabbit (control n = 6, amphotericin B n = 6, liposomal nystatin 3 mg/kg n = 6, liposomal nystatin 8 mg/kg n = 7).

We first confirmed the in vitro efficacy for H99 of the liposomal nystatin by formal MIC testing. The susceptibility testing indicated an MIC_100_ of 0.39 µg/mL, which is well below the published cryptococcal MIC_90_ [5]. Next, in order to assess the effect of liposomal nystatin on fungal burden in vivo, we infected the corticosteroid-treated (5 mg/kg, i.m., daily) rabbits and treated them with daily intravenous liposomal nystatin at either 3 mg/kg or 8 mg/kg, starting ~48 h after the infection, in two independent experiments. We used amphotericin B deoxycholate at 1 mg/kg as a positive control treatment. Furthermore, compared to the untreated rabbits, the rabbits treated with either 3 or 8 mg/kg of liposomal nystatin did not produce a significant reduction in the fungal burden in the CSF. Compared to the 3–6 log_10_ CFU/mL of yeast reductions seen in the CSF with amphotericin B treatment, the liposomal nystatin had little to no apparent treatment effect (Figure 1).

**Figure 2:**
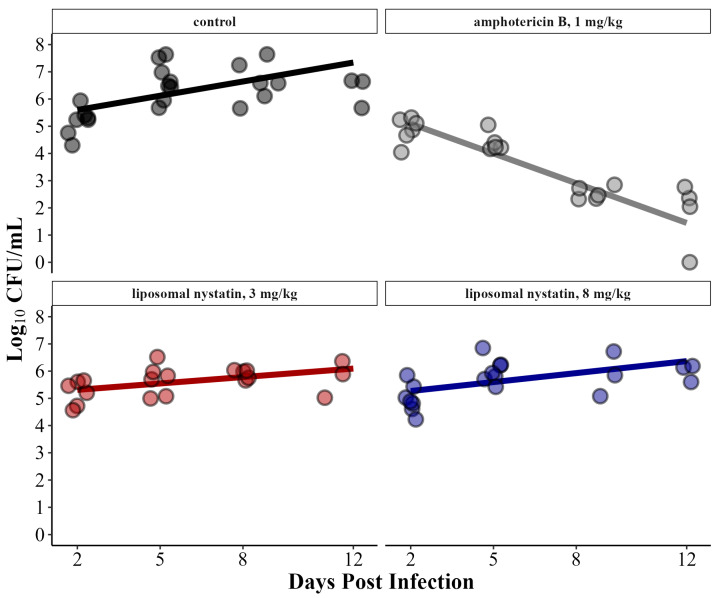
Individual data from serial CSF collections are represented by the dots. The lines were generated by calculating the estimated marginal means from a linear mixed effects model and extracting the slopes which represent the EFA.

To confirm the results seen with fungal burden over time, we calculated the EFA within each group using a linear mixed effects model. We extracted the slopes from the estimated marginal means of the model for each group (Figure 2). The untreated rabbits had a slope of +0.17 log_10_ CFU/mL/day, indicating an increase in fungal burden over time. Rabbits treated with liposomal nystatin at 3 mg/kg had a slope +0.08 log_10_ CFU/mL/day and rabbits treated with 8 mg/kg had a slope of +0.11 log_10_ CFU/mL/day, indicating no effect of the therapy, and in fact, a small increase in fungal burden over time occurred on treatment (Table 1). By comparison, rabbits treated with amphotericin B at 1 mg/kg had an EFA of −0.36 log_10_ CFU/mL/day, indicating a strong daily reduction in fungal burden over time. The consistent lack of a negative EFA in animals clearly indicates that liposomal nystatin did not have a therapeutic effect in this model, but amphotericin B did.

Given the consistent negative results with liposomal nystatin, we investigated the concentrations of nystatin in the plasma, brain, and cerebrospinal fluid collected from a random selection of rabbits in the studies to see if the drug reached the target tissue. The CSF and brain were collected at the completion of the study, approximately 24 h after the final dose. Levels of nystatin in the CSF were below the limit of detection for all six rabbits which were dosed with 3 mg/kg/day (Table 2). The rabbits treated with 8 mg/kg/day mostly had levels below the limit of detection (3 out of 5) 24 h after the last dose. These results match previously published results which reported CSF levels below the limit of detection for rabbits dosed with 2, 4, or 6 mg/kg/day, collected 30 min after the dose [12]. In the brain tissue, rabbits treated with 3 mg/kg/day had very low levels of nystatin (0.04 µg/g), but this was only tested in two rabbits. The concentration in the brain tissue of three rabbits dosed with 8 mg/kg/day was 1.67 (+0.88) µg/g. The plasma concentrations were 11.38 (+2.1) µg/mL for rabbits treated with 3 mg/kg, collected 30 min after dosing, and 24.89 (+7.93) µg/mL for rabbits treated with 8 mg/kg, collected 4 h after dosing. These results are comparable to the levels reported in the literature for uninfected rabbits, which were 6.09 (±0.86) µg/mL for rabbits treated with 2 mg/kg and 34.74 (±3.74) µg/mL for rabbits treated with 6 mg/kg collected 30 min after dosing [14]. These results indicate that the drug was able to reach the central nervous system but may not have been able to accumulate in sufficient quantities within the CSF during treatment, which would explain the negative antifungal therapeutic results.

## 4. Discussion

We demonstrated that liposomal nystatin is not effective for the treatment of CM in the rabbit model at 3 or 8 mg/kg. In these studies, we infected immune-suppressed rabbits with 10^8^ yeast cells of *C. neoformans*. The inoculum was higher than some of our recent studies, in which we used 10^6^ cells as the inoculum. However, despite the difference in starting inoculum sizes, the untreated controls and the amphotericin B positive controls had EFAs which were very similar to studies using the lower inoculum [15]. Indeed, the EFAs seen here for the positive and negative controls are very similar to results we published when testing other antifungal therapeutics [15]. This supports the reliability of the model and the robustness of the therapeutic system. However, we cannot eliminate the possibility that a lower inoculum may have shown some efficacy.

On the other hand, compared to amphotericin B, nystatin is not as effective in vivo. Nystatin is primarily used for oral candidiasis and dermatophytic infections. Efforts to use liposomal nystatin for systemic aspergillosis resulted in positive findings suggesting efficacy in two smaller clinical studies, although less robust than the evidence of efficacy observed from the animal models with other fungi [16,17]. We have tested the efficacy of many compounds in this rabbit CM model. The model has proven to be a very reliable and consistent predictor of eventual clinical efficacy in humans. Most significantly, the model provided strong preclinical evidence for the efficacy of short-duration dosing of lipid formulations of amphotericin B for the treatment of CM [18]. These results in rabbits using higher doses of lipid formulations were confirmed as a therapeutic approach in the AMBITION trial, and the formulation has become one of the primary approaches for antifungal treatment during the induction phase of therapy [4]. In this study, we unfortunately demonstrated that liposomal nystatin with high doses did not have an effect on the fungal burden in the rabbit model of CM. These results in the rabbit CM model were matched with the negative results of a clinical trial in which liposomal nystatin was tested for efficacy in CM [12]. Critically, these results demonstrate the predictive power of the rabbit CM model for efficacy in human clinical trials.

Liposomal nystatin’s failure may be explained by its pharmacokinetics. Our limited assessment of the drug distribution indicated that liposomal nystatin dosing at 3 mg/kg or 8 mg/kg in this model did not lead to detectable concentrations of the drug in the CSF. These results were consistent with published results in uninfected animals [14]. Despite low levels in the CSF, we did observe plasma concentrations which were above the reported MIC_90_ of 8 µg/mL for *C. neoformans*, and levels which were measured in the brain tissue were above the MIC_100_ value we observed for H99, 0.39 µg/mL, at both doses we tested. We did not quantify the fungal burden in the brain tissue, but it is possible that the fungal burden of the rabbits treated with liposomal nystatin was lower than the fungal burden of the untreated rabbits. Given that nystatin is a polyene, it is important to consider these concentrations relative to amphotericin B, a highly effective polyene antifungal agent. In previously published results looking at amphotericin B in this CM model, concentrations in the CSF were 0.003–0.006 µg/mL 30 min to 6 h after dosing [19]. These levels are well below the amphotericin B MIC_50_ for *C. neoformans* of 1–2 µg/mL. . Another explanation is that even amphotericin B has very low drug levels in the CSF in this model yet still has potent antifungal activity, likely due to its immunological effects, such as macrophage activation. Previously, we found that lipid products of amphotericin B were not as potent as amphotericin B deoxycholate as a second signal for macrophage activation [20]. Thus, liposomal nystatin’s poor antifungal activity in the CNS may be a combination of poor pharmacokinetics in the subarachnoid space; further, the liposomal formulation may have abrogated the polyene’s second signaling for macrophage activation and yeast killing. It is clear that polyenes, despite their broad in vitro anticryptococcal activity, must have each new formulation tested for efficacy against *Cryptococcus* in a robust CNS rabbit model prior to their testing in human clinical trials.

Recently, there has been renewed interest in using nystatin as an antifungal therapeutic [21]. Given the need for new antifungal therapies [22], optimization of liposomal nystatin may be a reasonable approach, given the large body of data on its use. Indeed, changes to the liposomes of the compound showed a robust anti-cryptococcal effect in the brain tissue in a murine model [23]. The negative results we obtained in this study could be related to the preferential tissue compartment of the model. In the rabbit model, the organism is inoculated directly into the central nervous system. Pharmacokinetic data using doses up to 8 mg/kg/day revealed concentrations below the limit of detection or barely detectable ones in the CSF, which is the tissue we assessed for fungal burden; the same measurements were used in early human clinical trials which similarly showed the drug’s failure [4,24]. In summary, liposomal nystatin was ineffective in the rabbit CM model, and these preclinical results matched the poor efficacy seen in human clinical trials, demonstrating both the utility and robustness of the rabbit model as a predictor of efficacy for humans and the challenges for using liposomal nystatin, despite its strong in vitro activity, as an agent for the treatment of CM.

## Figures and Tables

**Table 1:** Effective fungicidal activity.

Experimental Group	Number of Rabbits	Number of CSF Data Points	Slope (EFA) (log_10_ CFU/mL/day)	SE	Lower Limit 95% CI	Upper Limit 95% CI
control	6	24	0.17	0.05	0.08	0.26
amphotericin B, 1 mg/kg	6	21	−0.36	0.04	−0.45	−0.28
liposomal nystatin, 3 mg/kg	6	20	0.08	0.05	−0.02	0.17
liposomal nystatin, 8 mg/kg	7	20	0.11	0.05	0.01	0.21

**Table 2:** Nystatin drug levels.

Group	Time	CSF (mean ± SD) n *	Plasma (mean ± SD)	Brain (mean ± SD)
liposomal nystatin, 3 mg/kg	0.5	NA	11.38 (±2.1) n = 4	NA
liposomal nystatin, 8 mg/kg	4	NA	24.89 (±7.93) n = 3	NA
liposomal nystatin, 3 mg/kg	24	0 (+0) n = 6	0.01 (±0.02) n = 4	0.04 (±0) n = 2
liposomal nystatin, 8 mg/kg	24	0.02 (+0.03) n = 5	0.18 (±0.09) n = 3	1.67 (±0.88) n = 3

Measurements in µg/mL. * A value of 0 was used for measurements below the limit of quantification.

## Data Availability

Data will be shared upon request.
